# Tumor-Associated Microenvironment of Adult Gliomas: A Review

**DOI:** 10.3389/fonc.2022.891543

**Published:** 2022-07-07

**Authors:** Vincenzo Di Nunno, Enrico Franceschi, Alicia Tosoni, Lidia Gatto, Stefania Bartolini, Alba Ariela Brandes

**Affiliations:** ^1^ Department of Oncology, Azienda Unità Sanitaria Locale (AUSL) Bologna, Bologna, Italy; ^2^ Nervous System Medical Oncology Department, Istituto di Ricovero e Cura a Carattere Scientifico (IRCCS) Istituto delle Scienze Neurologiche di Bologna, Bologna, Italy

**Keywords:** microenvironment, glioma, oligodendroglioma, astrocytoma, H3K27 altered glioma, midline glioma, IDH wild type glioma

## Abstract

The glioma-associated tumor microenvironment involves a multitude of different cells ranging from immune cells to endothelial, glial, and neuronal cells surrounding the primary tumor. The interactions between these cells and glioblastoma (GBM) have been deeply investigated while very little data are available on patients with lower-grade gliomas. In these tumors, it has been demonstrated that the composition of the microenvironment differs according to the isocitrate dehydrogenase status (mutated/wild type), the presence/absence of codeletion, and the expression of specific alterations including H3K27 and/or other gene mutations. In addition, mechanisms by which the tumor microenvironment sustains the growth and proliferation of glioma cells are still partially unknown. Nonetheless, a better knowledge of the tumor-associated microenvironment can be a key issue in the optic of novel therapeutic drug development.

## Introduction

Gliomas are the most frequent primary tumors of the central nervous system (CNS) with an estimated incidence of 7.1/100,000 cases in the United States (1). Glioblastoma (GBM) encounters 55% of all glioma diagnoses while the remaining 45% of cases are represented by other glioma subtypes (1). Overall, gliomas are mainly divided into isocitrate dehydrogenase (IDH) mutant and IDH wild-type (wt) tumors (1–3). In the primary group, composed of IDH-mutated tumors, the World Health Organization (WHO) 2021 classification recognizes oligodendroglioma (presenting 1p19q codeletion) and astrocytoma (without 1p19q codeletion) (4). These tumors can be further divided into WHO grade 2 (oligodendroglioma and diffuse astrocytoma) and 3 (anaplastic oligodendroglioma and anaplastic astrocytoma) gliomas.

Gliomas without IDH mutations are defined as IDH-wt gliomas. According to the WHO 2021 classification, the presence of IDH-wt and other molecular alterations such as TERT (Telomerase Reverse Transcriptase), EGFR (Epidermal Growth Factor Receptor), or gain of chromosome 7/loss of chromosome 10 allows defining these tumors as a molecular GBM (4). Gliomas with H3K27 alterations (27th amino acid of Histone 3) is a new entity of IDH-wt gliomas diagnosed in pediatric patients but occasionally also in adults (4).

The prognosis of patients with gliomas (5) is extremely variable, ranging from decades in low-grade IDH-mutated gliomas to a few months in IDH-wt tumors (6). The standard clinical therapeutic approach is represented by maximal safe surgical resection followed by radiation therapy and chemotherapy (2, 3, 6).

In the last few years, important improvements toward a better understanding of genetic and epigenetic pathways regulating glioma development and growth have been done. These mechanisms could explain the different clinical courses and evolution of these malignancies. Indeed, genomic and epigenomic alterations differ in each glioma subtype explaining the different histology, clinical course, and biological behavior. The tumor-associated microenvironment appears to be another key element influencing the development, progression, and clinical evolution of gliomas.

Indeed, the tumor microenvironment (TME) composition is manipulated directly by cancer cells; therefore, TME composition changes according to the different alterations expressed by tumors. On the other hand, TME can sustain tumor growth and development in different ways (7).

Interactions between TME and GBM have been largely evaluated (7), while few data are available on patients with IDH-mutated/wt low-grade gliomas.

In this review, we examine current knowledge toward TME surrounding IDH-mutated and IDH-wt glioma (excluding GBM). To better understand TME composition, we analyzed the genomic landscape of each tumor subtype. Finally, we investigated possible therapeutic strategies aimed to target TME. Our focus is mainly oriented on adult gliomas; thus, we exclude pediatric malignancies and GBM from this review.

## IDH-Mutated Gliomas

Microscopically IDH-mutated, 1p19q codeleted oligodendrogliomas appear as tumor cells with rounded nuclei, clear perinuclear halons, frail capillaries, and focal microcalcification (3, 4, 6). An increased number of mitoses, vascular proliferation, and necrosis is observed in CNS WHO grade 3 oligodendrogliomas (3, 4, 6).

IDH-mutated 1p19q non-codeleted astrocytomas spread with perineuronal, perivascular, or subpial patterns and present nuclear atypia and pleomorphism (**Table 1**). The higher tumor grade is associated with increased mitotic activity, angiogenesis, and necrosis (3, 4, 6).

**Table 1:** Summary of adult glioma clinical behaviors.

Name	Percentage of all non-GBM gliomas	IDH	1p19q	Grade	H3K27	TERTEGFR7 gain/10 loss	Median age at diagnosis	Prognosis	Treatment after surgery
Oligodendroglioma (3, 4, 6, 8–12)	19%–26%	Mutated	Codeleted	2	No	No	42–44	17.5 years	Follow-up or RT→CT (PCV preferred to TMZ)
Anaplastic oligodendroglioma (3, 4, 6, 8–12)	14%–20%	Mutated	Codeleted	3	No	No	48–48.5	11.2 years	RT→CT (PCV preferred to TMZ)
Diffuse astrocytoma (3, 4, 6, 8–12)	24%–26%	Mutated	Non-codeleted	2	No	No	36–37	8.5–11	RT→CT (PCV preferred to TMZ)
Anaplastic astrocytoma (3, 4, 6, 8–12)	10%–23%	Mutated	Non-codeleted	3	No	No	35–40	6.5-9.3	RT→CT (TMZ preferred to PCV)
IDH-wt glioma (3, 4, 6, 11–14)	6%–12%	Wild-type	Non-codeleted	2–3	No	No	44–46	Unknown	RT→CT (TMZ) could be considered
Molecular GBM (3, 4, 6, 11–14)		Wild-type	No-codeleted	2–3	No	Yes	44–46	9–24 months	RT→CT (TMZ) *OR* RT/CT→ (TMZ)
Midline Glioma (4, 14)	<5%	Wild-type	No-codeleted	2	Yes	No	Young adult	6–20 months	RT→CT (TMZ) could be considered

RT, radiation therapy; CT, chemotherapy; PCV, procarbazine, CCNU, vincristine; TMZ, temozolomide; GBM, glioblastoma; IDH, isocitrate dehydrogenase.

Oligodendrogliomas and astrocytomas represent 40%–45% and 50%–55% of all glioma diagnoses with an estimated survival ranging from 6.5 to over 15 years (6, 11–13).

Maximal safe surgical resection is the standard of care for these tumors (2, 3). In WHO grade 2 astrocytomas and oligodendrogliomas, follow-up after surgery is considered in low-risk patients while adjuvant radiation therapy (RT) followed by chemotherapy [temozolomide/TMZ (9) or procarbazine plus lomustine plus vincristine/PCV (10, 15)] is commonly used in high-risk patients (8, 9).

### Genomic Landscape of IDH-Mutated Gliomas

Oligodendroglioma and astrocytoma significantly differ in their genomic alterations (16).

In addition to IDH1 or IDH2 mutations and 1p19q codeletion, oligodendrogliomas frequently present mutations of TERT (96%), CIC (Capicua Transcriptional Repressor, 62%), FUBP1 (Far Upstream Element Binding Protein 1, 29%), and/or PI3K (phosphoinositide 3-kinase, 20%) with overexpression of NOTCH1 (Notch homolog 1, translocation-associated, 31%) genes (16). Notably, ATRX (X-linked nuclear protein) mutations are rare in oligodendrogliomas and are mutually exclusive with TERT since both these two genes target the lengthening telomeres (16).

Almost all astrocytomas present p53 (94%) alterations, ATRX (86%) loss, or CDKN2A/CDKN2B (cyclin-dependent kinase inhibitor 2A/2B, 10%) homozygous deletion (16, 17). Curiously, astrocytomas present often non-canonical IDH1 mutations that are associated with improved survival (18–20). Recently, a next-generation sequencing (NGS) analysis carried out on 432 patients with anaplastic astrocytomas enrolled in the CATNON trial revealed a prognostic role of some selected genes. In particular, amplification of PDGFR (platelet-derived growth factor receptor) genes, CDKN2A/CDKN2B homozygous deletion, and PI3K mutations were independently associated with worse prognosis in patients with anaplastic (WHO grade 3) astrocytomas (17).

Another improvement toward a better knowledge of glioma genomic assessment was carried out in a large study adopting single-cell RNA sequencing (21). Researchers were able to evaluate with high precision the single-cell expression silencing confounding factors related to intratumoral genetic heterogeneity and genomic analysis of TME that can pollute large NGS studies (16).

In this study, researchers were able to identify, within tumor specimens assessed, three specific cellular populations: two differentiated tumoral cells belonging to oligodendrogliomas or astrocytomas and an undifferentiated phenotype (21). Through this precise gene expression analysis, the authors highlighted a shared expression between undifferentiated cells from oligodendrogliomas and astrocytomas, suggesting a shared progenitor of these two entities. These progenitor cancer cells presented alterations of key transcription factors such as SOX4 (SRY-Box Transcription Factor 4), SOX11 (SRY-Box Transcription Factor 11), and TCF4 (Transcription Factor 4). The percentage of these alterations increased according to CNS grade and the number of recurrences (21).

IDH-mutated gliomas seem to originate from a shared progenitor stem cell. Early acquired alterations are IDH with or without 1p19q codeletion. These events drive subsequent genomic alterations and explain the differences in terms of gene expression and TME (see below) within these two subtypes (21).

The progenitor cell of oligodendrogliomas and astrocytomas has a transcriptional phenotype similar to progenitor neural cells. These neural lineages can differentiate toward astrocytic or oligodendrocyte-like tumoral cancer cells (21, 22). These findings overturned the previous belief that there were two distinct progenitor cells for oligodendrogliomas and astrocytomas (23).

Differently, the progenitor cells of IDH-wt gliomas assume high levels of cellular state plasticity. Indeed, these ancestral cells can differentiate toward different transcriptional subtypes named mesenchymal, classic, and/or proneural (24). Subsequent studies differentiated the transcriptional subtypes into four main groups named astrocyte (EGFR amplified), oligodendrocyte (PDGFRA amplified), neural progenitor (CDK mutated), and mesenchymal (NF1 mutated)-like lineages (25).

Another important issue is the absence of a defined hierarchy between tumoral subtypes within IDH-wt gliomas (25). Thus, cancer cells can proliferate and switch from one subtype to another with high plasticity and heterogeneity (25). This finding diverges from what was observed in IDH-mutated and IDH-wt H3K27 gliomas. Indeed, IDH-wt H3K27 altered gliomas present a specific progenitor cell that has an oligodendrocyte-like transcription lineage (26). During the evolution, these progenitor cells lose (due to the alterations of histone 3) oligodendrocyte lineage differentiating toward an astrocyte-like cancer cell phenotype (26).

### Microenvironment of IDH-Mutated Gliomas

**Figure 1:**
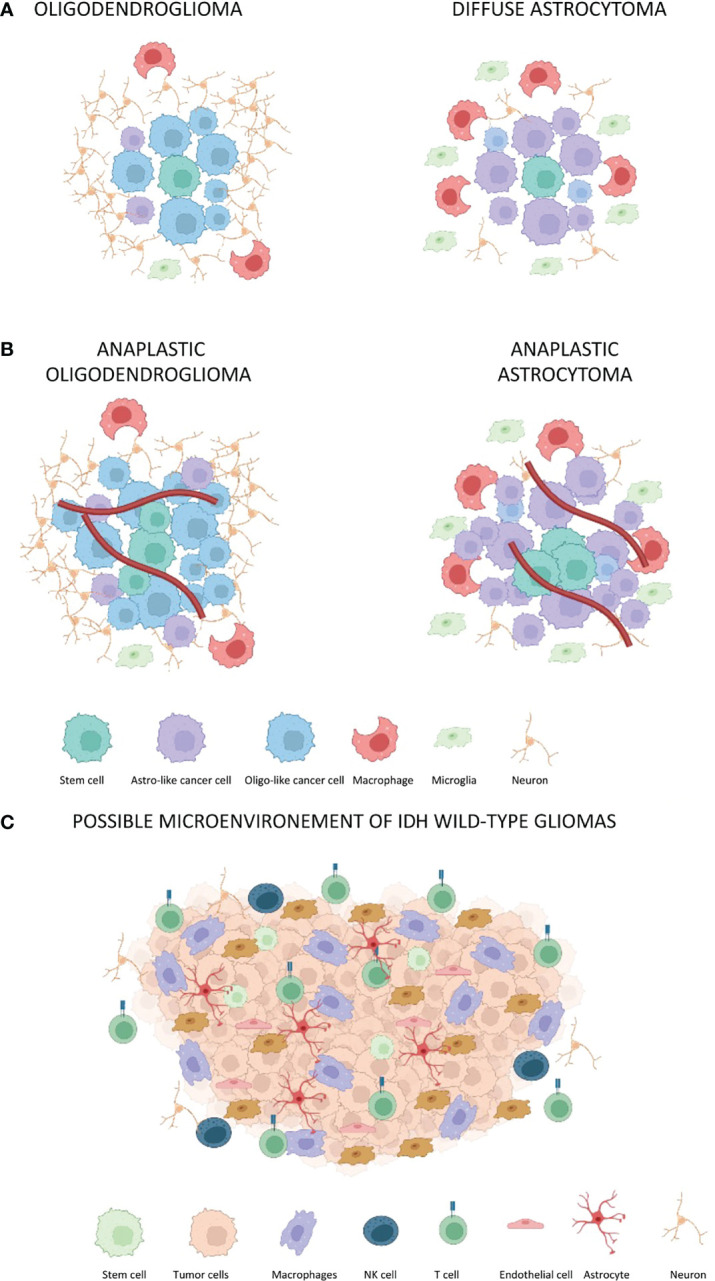
The tumor-associated microenvironment of IDH-mutated and IDH-wt gliomas. Red lines represent blood vessels. **(A)** Oligodendroglioma and diffuse astrocytoma without a significant blood vessel proliferation. The oligodendroglioma microenvironment presents a reduced percentage of macrophages, microglia, and astro-like cells as compared to diffuse astrocytoma. **(B)** The same composition of microenvironment associated with oligodendroglioma and astrocytoma is maintained in anaplastic gliomas. However, there is an increased tumor cell proliferation as well as increased angiogenesis. **(C)** Composition of microenvironment associated to GBM with CD4 immune regulatory and CD8 T-lymphocyte. Notably, CD8 lymphocytes assume the classical exhaustion phenotype expressing several inhibitory receptors including the PD-1, T-cell membrane protein 3 (TIM3), lymphocyte activation gene 3 protein (LAG3), and T-cell immunoreceptor with immunoglobulin and ITIM domains (TIGIT) (27).

Interactions between tumor cells and surrounding cells are complex and depend on the genetic expression of a specific tumor subtype (**Figure 1**). In general, tumor cells interact with immune cells, endothelial cells, and neurons (**Table 2**).

**Table 2:** The microenvironment of low-grade glioma.

Oligodendroglioma	Astrocytoma	H3K27 glioma	IDH-wt gliomas**
The microenvironment expression reflects a microglia signature (CX3CR1, P2RY12/13)* more than the macrophage one (21, 28–32).	Inflammatory expression following macrophage signature (CD163, TGFβ1, and F13A1) (21, 28–32).	Microglia assumes a specific morphology with enlarged cell bodies and shorter processes (33–35).	The environment is mainly composed of microglia and tumor-associated macrophages (7, 36).
Microglia signature is not associated with angiogenesis (21).	Macrophage signature is associated with angiogenesis (21).	Microenvironment enriched in microglia and macrophage concentration (33–35) with low lymphocytes	Recruitment of monocytes by secretion of CCL2-7, GDNF, TNF, CSF-1, and GM-CSF (7, 36).
Lower percentage of CD8+PD1+, CD4+ TIM3+, and regulatory T cells is associated with a less immune-suppressive stroma (37).	Increased percentage of CD8+PD1+, CD4+ TIM3+, and regulatory T cells is associated with an increased immune-suppressive stroma (37).	Macrophages associated with H3K27 gliomas present a lower expression of IL6, IL1A, IL1B, CCL3, and CCL4 (33–35). Increased percentage of CCL2, CCL5, CSF1, CXCL12, and PDGFA (14).	Macrophages are associated with immune-suppressive stroma mainly due to the secretion of TGFβ (7, 36).
Unknown interactions with neurons. Possible release of glutamate by SLC7A11, which interacts with NMDA receptor inducing calcium intake (38–40).		Unknown interactions with neurons.	Astrocytes switch from a physiological phenotype to tumor-initiating cells. Neurons can stimulate tumor growth by secretion of glutamate, PI3K, FAK, HSPA5, and neuroglin 3 (7, 36).

* CX3CR1 and P2RY12/13 could be associated with specific functions carried out by microglia regulating trophic functions and interactions with neurons (41).

** The majority of data about microenvironment composition in patients with IDH-wt gliomas are provided by studies investigating glioblastoma cancer cells.

CCL3-4, C-C motif chemokine ligand 3-4; CX3CR1, C-X-C motif chemokine receptor 1; CSF-1, colony-stimulating factor 1; FAK, focal adhesion kinase; F13A1, coagulation factor XIII A chain; GDNF, glial cell-derived neurotrophic factor; GM-CSF, granulocyte-macrophage colony-stimulating factor; HSPA5, health shock protein family A; IL6/1A/1B, interleukin 6; interleukin 1A; interleukin 1B; PD1, programmed death receptor 1; PI3K, phosphoinositide 3-kinase; P2RY12/13, purigenic receptor P2Y12/13; SLC7A11, solute carrier family 7 member 11; TGFβ1, transforming growth factor β1; TIM3, T-cell immunoglobulin and mucin-domaining containing-3; TNF, tumor necrosis factor.

Important insights into connections between IDH mutant gliomas and immune cells have been provided by the study carried out by Venteicher et al. investigating single-cell RNA expression (21).

The authors identified an inflammatory expression derived from two specific and different signatures (21). The first was a microglia signature characterized by specific markers such as CX3CR1 (C-X-C motif chemokine receptor 1), P2RY12 (purigenic receptor P2Y12), and P2RY13 (purigenic receptor P2Y13). The second signature was a macrophage signature identified by the expression of CD163, TGFβ1 (transforming growth factor β1), and F13A1 (coagulation factor XIII A chain) (21). Notably, the distribution of the signature markers of both microglia and macrophages followed a continuum more than a bimodal scheme. Therefore, a macrophage that has reached the TME can acquire a microglia-like expression according to the phenotype expressed by cancer cells (21). On the other hand, microglia cannot differentiate to a macrophage immune profile (21). Finally, a subtle but reported difference between resident and tissue-derived macrophages has been reported (21).

Other important findings of this study revealed that macrophage signature and expression were more frequently associated with astrocytoma as compared to oligodendroglioma (21). Moreover, the macrophage signature was significantly associated with angiogenesis and endothelial activities. The same association was not true in cells expressing microglia signature.

According to these results, the presence of macrophages seems to enhance angiogenesis, progression, and glioma development. However, the mechanisms behind these interactions are unknown (21).

In this optic, it is essential to observe that the role of macrophages on glioma proliferation and development has only been investigated with a single-cell approach; thus, no definitive conclusions can be deduced. This is mainly because mechanisms beyond macrophage and glioma proliferation stimulation are largely unknown.

A subsequent study investigated TME composition in patients with IDH-mutated gliomas. These studies adopted different approaches involving flow cytometry, RNA sequencing, protein arrays, culture assays, and spatial tissue characterization (42) or also high-dimensional single-cell profiling (43). These assessments revealed a disease-specific enrichment of immune cells with a significant difference in proportional abundance of microglia, macrophages, neutrophils, and T cells (42). In particular, macrophages showed a distinctive signature trajectory that differs according to the primary tumor subtype (43).

A similar interaction has also been observed in GBM. This confirms that the macrophage signature is associated with angiogenesis and tumor progression (28–32). GBM cancer cells can attract monocyte, microglia, and macrophage by the production of several factors including CCL2 (C-C motif chemokine ligand 2), CCL7 (C-C motif chemokine ligand 7), GDNF (glial cell-derived neurotrophic factor), SDF1 (stromal cell-derived factor 1), TNF (tumor necrosis factor), VEGF (vascular endothelial growth factor), ATP (adenosine triphosphate), CSF-1 (colony-stimulating factor 1), GM-CSF (granulocyte-macrophage colony-stimulating factor), and expression of OLIG2 (oligodendrocyte transcription factor 2). Once attracted into the TME, macrophages can attract monocytes by the production of CCL2 and CCR2. Monocytes can be further oriented toward a macrophage signature by factors secreted by GBM (7, 28–32).

Macrophages contribute to the development of an immune-suppressive environment by the production of TGFβ1, ARG1 (arginase 1), and/or IL-10 (interleukin 10) (44). Macrophages can further stimulate angiogenesis through the production of VEGF (vascular endothelial growth factor) and metalloproteases (44).

It is important to remark that all these interactions have been demonstrated in GBM while no data are available on low-grade IDH-mutated gliomas.

A recent study investigating transcriptomic data of low-grade gliomas associated with TME identified three specific immune signatures (45). The first signature (Im1) identified a high number of T cells, Th17, and mast cells. The second signature (Im2) was composed of macrophages and exhausted CD8+ T cells. Tumors harboring the Im2 signature were associated with worst prognosis. Finally, the third signature (Im3) was composed of T-helper, antigen-presenting cells, and macrophages; 23.7% of tumor samples analyzed in this study were IDH-wt tumors; therefore, it is unknown which patterns are present in IDH mutant low-grade gliomas (45).

Another trial (37) assessed the single-cell expression of TME associated with 10 WHO grade 2 astrocytomas and 4 WHO grade 2 oligodendrogliomas. This study identified the presence of several immune cells like CD8+, CD4+, regulatory T cells, and natural killer cells (37). This is one of the first studies characterizing tumor-associated lymphocytes in low-grade gliomas. Notably, the authors identified that TME associated with astrocytoma presented a more inhibitory feature as compared with those observed in oligodendrogliomas. Finally, the increased percentage of CD8+ PD-1 (programmed death receptor 1) expressing T cell and CD4+ TIM3 (T-cell immunoglobulin and mucin-domaining containing-3) expressing T cell in association with regulatory T cells and macrophages was mainly responsible for an immune-suppressive contexture (37).

Interactions between IDH-mutated glioma cells and neurons and normal glial cells are largely unknown. It is well known that glioma is clinically associated with several neurological symptoms including cognitive or motility deficits, verbal fluency, headaches, and seizures (46–48). Notably, before diagnosis, low-grade gliomas often have a pre-symptomatic period. At this time, tumors may occupy a significant volume of the brain without manifesting significant symptoms. It has been proposed that neurons and glial cells can adapt to the tumor presence by activating a reactive response resulting in plasticity (49–51). There are several remarkable examples of this plasticity (50).

Brain tissue could adapt to the presence of tumors even if the mechanisms of this plasticity remain largely hidden. Furthermore, we ignore if low-grade IDH-mutated tumor cells can directly interact with neurons and altered glial cells. Tumor cells produce glutamate, which is released through the SLC7A11 (solute carrier family 7 member 11) glutamate–cysteine exchanger (38–40). The glutamate excess interacts with NMDA (N-methyl-D-aspartate receptor) receptors, inducing an influx of calcium on neurons and resulting in seizure onset and neuron death (38–40). Astrocytes activated after an injury can promote the secretion of cytokines and growth factors (52). Furthermore, astrocytes can also alter the permeability of the blood–brain barrier (BBB) (52). It has been demonstrated that GBM cells use astrocyte activation to sustain tumor growth and development while this has not been demonstrated in low-grade IDH-mutated tumors. Curiously, a recent study recognizes the importance of the glucose transporter GLUT1, which seems to mediate glioma cells’ perineuronal satellitosis in mice (53).

The study of the interactions between low-grade IDH-mutated tumor cells and surrounding neurons/glial cells is one of the most attractive and emerging issues. Future studies will probably offer novel insights into these important connections. Notably, it has been demonstrated that GBM interacting with neurons in a specific niche can differentiate toward an oligodendrocyte subtype (54), losing their infiltrative behaviors. Neuron stimulation could therefore modify tumor cell proliferation and development, making these interactions of particular interest.

## IDH-wt Gliomas

The IDH-wt gliomas represent a heterogeneous family of tumors. In general, IDH-wt astrocytoma represent 5%–12% of all low-grade gliomas (6, 11–13). These tumors are diagnosed in older age (45–55 years) compared to IDH-mutated gliomas and are associated with shorter survival (from 15 to 36 months) (6, 11–13).

No randomized trials have been designed to assess the role of systemic treatments in IDH-wt WHO grade 2 or 3 tumors (2, 3). A second interim analysis of the CATNON trial showed in a subgroup analysis that neither concurrent nor adjuvant TMZ was associated with a survival improvement in this population (8). To date, the most appropriate clinical management after surgical resection of IDH-wt gliomas (excluding GBM) is unclear.

The diffuse midline H3K27M glioma is a novel entity recognized by the WHO 2021 classification (4). This tumor involves 80% of brain stem tumors in children and adolescents with an estimated median survival of 9–11 months. Occasionally, these tumors can also be diagnosed in adult patients and are associated with the same dismal prognosis observed in children (1). To date, no standard therapeutic approaches for midline gliomas have been approved, and inclusion in clinical trials should be encouraged (3).

### Genomic Landscape of IDH-wt Gliomas

The TCGA assessed 282 low-grade gliomas, of which 56 (19.8%) were low-grade IDH-wt subtypes. In this study, the most frequent mutated genes were as follows: TERT (64%), EGFR (27%), PTEN (23%, phosphatase and tensin homolog), NF1 (20%, neurofibromin 1), TP53 (14%), and PIK3CA (9%) (16). Focal deletions of CDKN2A and RB1 (retinoblastoma-associated protein 1) occurred in 63% and 25% of cases, respectively. Gain of chromosome 7/loss of chromosome 10 was evident in 56% of cases (16).

As previously reported, IDH-mutated tumors could share a common progenitor that can differentiate into an astrocytic or oligodendroglioma phenotype (21). IDH-wt tumors are composed of cancer cells with the ability to modify their transcriptional profile assuming cellular plasticity (24, 25, 55, 56) and assuming all transcriptional subtypes (57) exchange within the same tumors (24, 25, 56). In other words, IDH-wt cancer cells can modify their phenotype ranging from the following subtypes:

1) Astrocytic-like cells characterized mainly by EGFR amplification,
2) Oligodendrocyte/neural progenitor phenotype frequently associated with PDGFRA/CDK4 amplification, and
3) Mesenchymal transcriptome associated with NF1 mutation.

The complexity of this scheme makes it clear how IDH-wt can be heterogeneous and therefore extremely similar to GBM (25).

It is important to remark that all these data have been provided by patients harboring an IDH-wt GBM while no studies focused on precursor cells within low-grade IDH-wt gliomas.

### Genomic Landscape of IDH-wt H3K27 Midline Gliomas

In midline gliomas, the pathognomonic alterations are represented by the lysine-to-methionine substitution at position 27 of histones 3.1 and 3.3 (58–61).

These alterations lead to the inactivation of the PRC2 (polycomb repressive complex-2 methyltransferases complex) hiding gene expression (58–61). This alteration is diagnosed in about 80% of cases of midline glioma. Nonetheless, other mechanisms converge to PRC2 altered function (4). Indeed, the EZH inhibitory protein (EZHIP, CXorf7) is overexpressed in some midline gliomas and posterior fossa type A ependymomas (62). The hyperexpression of EZHIP leads to an inhibitory activity on PCR2 similar to H3 mutations (62). These tumors often present p53 (42%), ACVR1 (activin A receptor type 1), PPM1D (9%–23%, phosphatase Mg2+/Mn2+-dependent 1D), and PI3K mutations (58–61, 63, 64). Notably, PPM1D mutations are mutually exclusive with p53 (activation of PPM1D leads to p53 inactivation) (65). Other amplified genes are PDGFB (platelet-derived growth factor B), CCND1 (cyclin D1), CCND2 (cyclin D2), CCND3 (cyclin D3), CDK4 (cyclin-dependent kinase 4), and CDK6 (cyclin-dependent kinase 6) (58–61, 63, 64).

Precursor cells of diffuse midline gliomas display an oligodendrocyte phenotype (26). Nonetheless, the alteration occurring in PRC2 blocks the differentiation toward oligodendrocyte subtype; thus, the cancer cells assume an astrocyte-like phenotype instead of an oligodendrocyte one (26).

### Microenvironment of IDH-wt Gliomas

Before the 2021 WHO classification, which recognized the diagnosis of “molecular glioblastoma”, IDH-wt gliomas were considered distinct tumor subtypes (66). In a study published in 2015 by Reuss et al. evaluating the molecular assessment of 160 IDH-wt glioma specimens, almost all tumors were finally classified as GBM or midline glioma (66). Not surprisingly, studies assessing TME on IDH-wt gliomas present the same results observed in similar analyses carried out on GBM patients (7, 67). In 2020, a study assessed and validated a specific immune signature in cohorts of patients with low-grade IDH wild-type gliomas (68). The authors identified an immune signature associated with a worse prognosis and characterized by high expression of immune-exhaustion markers and immune-depressive cytokines released by macrophages (68). On the other hand, researchers also identified a second phenotype associated with an elevated expression of lymphocyte and plasma cell-related genes (68).

In conclusion, most studies assessing TME on IDH-wt gliomas assessed the TME composition of GBM (7, 67). The complex interactions between tumor cells and TME (42, 43, 69, 70) required a specific and detailed discussion and are outside the scope of the current paper.

There are few studies investigating the TME of patients with midline gliomas. Nonetheless, there are particular issues concerning the tumor immune-associated stroma of these malignancies. These are immunologically cold tumors with a low percentage of T/NK cells (14). The main factors expressed are CCL2, CCL5, CSF1, CXCL12, and PDGFA with mainly enhanced microglia surrounding tumor cells (14).

It has been demonstrated that microglia associated with midline glioma assume their morphology with enlarged cell bodies and shorter processes (33–35). Similar to IDH-mutated astrocytomas and GBM, the microenvironment of midline gliomas is also enriched with macrophages. The PDGFB seems to be mainly responsible for macrophage recruitment (33–35). Compared to other CNS primary tumors, macrophages associated with midline gliomas have a lower expression of IL6, IL1A, IL1B, CCL3, and CCL4. Of interest, a gene-expression study demonstrated a significant difference between macrophages associated with GBM and midline gliomas (35). In particular, GBM-associated macrophages express mainly genes related to monocyte/neutrophil chemotaxis and chemokine while midline glioma-associated macrophages express genes related to angiogenesis, extracellular matrix organization, and angiogenesis (33–35). Furthermore, these tumors present a non-inflammatory environment as demonstrated by a low concentration of natural killer cells and infiltrating lymphocytes. This inflammation-desert microenvironment can be partially attributed to the reduced presence of key cytokines including the IL2 resulting from high levels of TGFβ and IL8 (14, 34). The other two key factors commonly observed in midline gliomas are a high concentration of the chemokines CCL2, CCL5, and the receptor PDGFRA, which directly support proliferation and cell survival (71).

While the midline glioma-associated immune contexture has just begun to be investigated, there is no data regarding interactions of these cells with other elements such as neurons, white matter, and glial cells.

In brief, GBM TME has a large infiltration of immune cells; however, their immune activity is mainly shifted toward an immune-suppressive phenotype. Midline glioma TME presents unique macrophages and microglia with its characteristics and morphology. Midline glioma shows a reduced presence of inflammation cells rather than a high number of immune cells with an inhibited response as observed in GBM TME.

## Therapeutic Implications and Future Developments

The TME offers novel targets for the treatment of malignant gliomas (72–74). The clinical importance of TME has been largely investigated in GBM (7), while only recently has it been assessed in patients with low-grade gliomas. Furthermore, the majority of studies concentrate their investigation on the immune cells’ composition of TME excluding other important elements such as neurons, stromal cells (such as fibroblasts), and other glial cells (75).

Among patients with IDH-mutated gliomas, the importance of tumor-associated cells is gaining increasing interest; however, no therapeutic drugs are acting directly on TME.

There are several systemic agents shown to modify the TME (76).

Immune checkpoint inhibitors (ICIs) are agents able to restore a suppressed immune response against tumors by targeting specific receptors named immune checkpoints. The programmed death receptor 1 (PD1) is the target of the monoclonal antibodies nivolumab and pembrolizumab, which have been tested in patients with GBM. In GBM, pembrolizumab has been investigated as a neoadjuvant treatment shown to induce a significant modification of the TME. These modifications consisted of an increased number of T cells, reduced monocytic population, activation of interferon γ-related gene expression, and downregulation of cell-cycle-related genes (77). Similarly, also the PD-1 inhibitor nivolumab can modulate the TME composition of GBM patients (78). Despite these positive results, no trials demonstrated that ICIs improved the clinical outcome of patients with GBM (79). To date, nivolumab is under evaluation in combination with the IDH inhibitor ivosidenib in patients with IDH-mutated tumors (NCT04056910).

Vaccines are other treatment strategies employed for the treatment of patients with gliomas (80). The NOA16 trial was a phase I study testing an IDH1-specific peptide vaccine among patients with IDH1-mutated gliomas. Notably, of the 33 patients receiving the vaccine, 93.3% presented an immune response (81). Patients responding to the vaccine more frequently had a pseudo-progression suggesting inflammation on the tumor site. Moreover, these same patients showed an increased T-cell response switching from an immune-inhibited to an immune-active environment (81). IDH vaccines are under evaluation in patients with gliomas (NCT03893903).

IDH inhibitors are a promising treatment approach (82), and a phase III trial (INDIGO, NCT04164901) is currently assessing the IDH pan-inhibitor vorasidenib. Modifications of TME during IDH inhibition are unknown and could be an interesting issue to investigate in case of a successful approval of these drugs.

Differently, manipulation of the TME is an emerging treatment strategy in patients with IDH-wt gliomas and especially H3K27 altered diffuse midline gliomas.

Peptide vaccines targeting the H3K27 mutation as well as dendritic cell vaccines have been investigated in the pre-clinical model (83, 84). In particular, peptide vaccine was shown to improve immune response against tumors shifting from an immune-suppressive microenvironment to an active one.

Phase I studies (NCT03396575 and NCT02960230) are currently investigating vaccines in this setting.

Other strategies that aimed to switch the immune microenvironment of midline gliomas consist of oncolytic virus (NCT02960230) and ICIs (NCT03330197 and NCT02359565). Engineered T lymphocytes (CAR T cells) are a new class of compounds consisting of lymphocytes obtained from patients, amplified and activated against tumor cells artificially, and then reinjected into the patients (85). This approach has been successfully tested on hematological malignancies and will also be evaluated in solid tumors. A phase I study employing HER 2 oriented CAR T cells showed promising activity in midline gliomas and a safety profile (86). There are some phase I trials evaluating this approach on patients with newly diagnosed (NCT04099797, NCT04196413, and NCT04185038) and recurrent (NCT04099797) midline glioma.

IDH-wt diffuse gliomas represent a heterogeneous class of tumors. It is reasonable to suppose that these tumors display a similar TME of GBM and thus could benefit from strategies aimed to target angiogenesis as well as an immune response against tumors (7). To date, there are no trials investigating agents modifying TME tailored for patients with IDH-wt diffuse gliomas.

The study of microenvironment composition is an emerging issue in patients with low-grade gliomas. In the majority of cases, we have only limited data about its composition according to tumor subtype. On the other hand, improved knowledge of this key element would improve the clinical management of these tumors in different possible ways. For example, microenvironment composition could be an element associated with prognosis and/or different responses to treatment provided. In this optic, the microenvironment could be assessed as a prognostic or predictive factor independently associated with clinical outcomes. A deeper knowledge of microenvironment composition would also improve the selection of patients to enroll in clinical trials (which is essential considering the rarity of the diseases and the long follow-up required for final data). Targeting microenvironment composition could also be a promising therapeutic option. Due to the approval of ICIs, the composition of immune cells surrounding tumors is the most assessed issue. Nonetheless, interactions between neurons, glial cells, endothelial cells, and stromal cells could hide important potential targets for therapy.

## Conclusion

Few studies investigated the TME of patients with glioma (excluding GBM). The composition of TME differs according to the genomic expression and mutations exhibited by cancer cells and can be modified by systemic treatments. Vaccines built with IDH1 and H3K27 peptides could be an interesting approach as these agents showed to modify microenvironment composition and improve immune response against tumors. A combination of different agents can further amplify the effect on TME resulting in improved anti-tumor activity.

## Author Contributions

VN and LG: writing and draft. EF, AB, AT, and SB: project conception and reviewing. All authors contributed to the article and approved the submitted version.

## Conflict of Interest

The authors declare that the research was conducted in the absence of any commercial or financial relationships that could be construed as a potential conflict of interest.

## Publisher’s Note

All claims expressed in this article are solely those of the authors and do not necessarily represent those of their affiliated organizations, or those of the publisher, the editors and the reviewers. Any product that may be evaluated in this article, or claim that may be made by its manufacturer, is not guaranteed or endorsed by the publisher.

## Abbreviations

ACVR1, Activin A receptor type 1; ARG1, Arginase 1; ATP, Adenosine triphosphate; ATRX, X-linked nuclear protein; CCL2, C-C Motif Chemokine Ligand 2; CCL3, C-C Motif Chemokine Ligand 3; CCL4, C-C Motif Chemokine Ligand 4; CCL7, C-C Motif Chemokine Ligand 7; CCND1, Cyclin D1; CCND2, Cyclin D2; CCND3, Cyclin D3; CDK4, Cyclin-dependent kinase 4; CDK6, Cyclin-dependent kinase 6; CDKN2A/CDKN2B, cyclin-dependent kinase inhibitor 2A/2B; CIC, Capicua Transcriptional Repressor; CNS, Central nervous system; CX3CR1, C-X-C Motif Chemokine Receptor 1; CSF-1, Colony-stimulating factor 1; EZHIP, EZH inhibitory protein; F13A1, Coagulation Factor XIII A Chain; FUBP1, Far Upstream Element Binding Protein 1; EGFR, Epidermal Growth Factor Receptor; GBM, Glioblastoma; GDNF, Glial Cell-Derived Neurotrophic Factor; GM-CSF, Granulocyte-Macrophage Colony-Stimulating Factor; H3K27, 27th amino acid of Histone 3; H3G34, 34th amino acid of Histone 3; IDH, Isocitrate Dehydrogenase; IL-1A/B, Interleukin 1A/1B; IL-6, Interleukin 6; IL-10, Interleukin 10; NF1, Neurofibromin 1; NGS, Next-Generation Sequencing; NMDA, N-methyl-D-aspartate receptor; NOTCH1, Notch homolog 1, translocation-associated; OLIG2, Oligodendrocyte transcription factor 2; RT, Radiation Therapy; P2RY12, Purigenic Receptor P2Y12; P2RY13, Purigenic Receptor P2Y13; P53, Tumor suppressor protein 53; PCV, Procarbazine, lomunstine, vincristine; PD-1, Programmed Death Receptor 1; PDGFA, Platelet-derived growth factor subunit A; PDGFB, Platelet-derived growth factor subunit B; PDGFR, Platelet-Derived Growth Factor Receptor; PI3K, Phosphoinositide 3-kinase; PPM1D, Phosphatase Mg2+/Mn2+ dependent 1D; PRC2, Polycomb repressive complex-2 methyltransferases complex; PTEN, Phosphatase and tensin homolog; RB1, Retinoblastoma-associated protein; SLC7A11, Solute Carrier Family 7 Member 11; SDF1, Stromal cell-derived factor 1; SOX4, SRY-Box Transcription Factor 4; SOX11, SRY-Box Transcription Factor 11; TCF4, Transcription Factor 4; TERT, Telomerase Reverse Transcriptase; TGCA, The Cancer Genome Atlas; TGFβ1, Transforming growth factor β1; TIM3, T-cell immunoglobulin and mucin-domaining containing-3; TME, Tumor microenvironment; TMZ, Temozolomide; TNF, Tumor necrosis factor; VEGF, Vascular Endothelial Growth Factor; WHO, World Health Organization;
